# Balancing strength and vulnerability: A qualitative study on the mental health impact of the Superwoman Schema on African American female dementia caregivers

**DOI:** 10.1002/alz70858_103382

**Published:** 2025-12-24

**Authors:** Shanae Lakel Rhodes, Pamela Recto

**Affiliations:** ^1^ UT Health San Antonio, San Antonio, TX, USA

## Abstract

**Background:**

Existing literature presents a health paradox among African American caregivers, who often report worse physical health but better mental health related to caregiver burden compared to other racial groups. However, inconsistencies exist across the research on mental health outcomes for this population. This study aimed to explore how Superwoman characteristics and caregiver burden influence the mental health of African American female caregivers, offering a deeper understanding of their unique experiences.

**Method:**

A qualitative descriptive approach, incorporating ethnographic strategies, was used to conduct 15 individual interviews via Zoom, generating 1,012 minutes of transcribed data. This data provided valuable insights into the lived experiences of African American women caregivers. The analysis was guided by Woods‐Giscombe's Superwoman Schema (SWS) as a sensitizing framework. A mid‐level analysis of the data led to the identification of 13 emerging themes.

**Result:**

The findings highlighted both the challenges and resilience of African American women caregivers. Barriers identified included *family discrepancies & trauma*, *intracultural expectations*, and *the impact of historical and ongoing oppression/societal barriers*, including racism and trauma. However, the data also illuminated sources of resilience among African American women caregivers, including *prayer/faith in God*, *self‐prioritization*, and *cultural dignity/strength of the Black woman*.

**Conclusion:**

These findings offer a comprehensive understanding of the complex experiences of African American women caregivers, providing insights into the adversities they face and the strengths they draw upon. Such insights are essential for developing targeted, culturally sensitive interventions to support this underserved population.

